# Combining GWAS, Genome-Wide Domestication and a Transcriptomic Analysis Reveals the Loci and Natural Alleles of Salt Tolerance in Rice (*Oryza sativa* L.)

**DOI:** 10.3389/fpls.2022.912637

**Published:** 2022-06-16

**Authors:** Yang Lv, Jie Ma, Hua Wei, Fang Xiao, Yueying Wang, Noushin Jahan, Mohamed Hazman, Qian Qian, Lianguang Shang, Longbiao Guo

**Affiliations:** ^1^State Key Laboratory for Rice Biology, China National Rice Research Institute, Chinese Academy of Agricultural Sciences, Hangzhou, China; ^2^Agricultural Genomics Institute at Shenzhen, Chinese Academy of Agricultural Sciences, Shenzhen, China; ^3^Department of Agronomy, Khulna Agricultural University, Khulna, Bangladesh; ^4^Agricultural Genetic Engineering Research Institute, Giza, Egypt

**Keywords:** rice, genome-wide association study (GWAS), salt tolerance, transcriptome analysis, domestication detection

## Abstract

Soil salinity poses a serious threat to the sustainable production of rice (*Oryza sativa* L.) throughout the world. Thus, the detection of loci and alleles responsible for salt tolerance is fundamental to accelerating the improvement of rice and producing the resilient varieties that will ensure future harvests. In this study, we collected a set of 191 mini-core rice populations from around the world, evaluated their salt tolerance based on plant growth and development phenotypes at the seedling stage, and divided a standard evaluation score (SES) of visual salt injury into five different grades. We used ∼3.82 million single nucleotide polymorphisms (SNPs) to identify 155 significant SNPs and 275 genes associated with salt sensitivity based on a genome-wide association study (GWAS) of SES. In particular, two candidate genes, *ZFP179* and *OsDSR2*, were associated with salt tolerance, and *OsHKT1;1* was co-detected in the entire GWAS of all the panels and *indica*. Additionally, we investigated the transcriptional changes in cultivars 93-11 and PA64s under normal and salinity stress conditions and found 517 co-upregulated and 223 co-downregulated genes. These differentially expressed genes (DEGs) were highly enriched in “response to chemical” and “stress” based on the gene ontology enrichment analysis. Notably, 30 candidate genes that were associated with the salt tolerance analysis were obtained by integrating GWAS and transcriptomic DEG analyses, including 13 cloned genes that had no reports of tolerance to salt and 17 candidate genes whose functions were unknown. To further explore these genes and their alleles, we performed haplotype analysis, genome-wide domestication detection, and transcriptome analysis to breed improved varieties. This data and the genetic resources provided will be valuable for the development of salt tolerant rice varieties.

## Introduction

The levels of salinization in the soil are increasing significantly owing to improper irrigation and limited freshwater supplies. Soil salinity inhibits plant growth and development, metabolic changes, and ion sequestration and exclusion (Chen et al., 2018; Van Zelm et al., 2020). Consequently, salinization of the soil represents a considerable abiotic stress for agricultural production and the ecological environment, limits the utilization of arable land, and thus constitutes a global problem that represents the primary source of agricultural crisis in the world (Bailey-Serres et al., 2019; Hassani et al., 2020). In light of this, the utilization of naturally evolved traits and population genetics to accelerate crop improvement and develop resilient production systems is necessary to ensure the viability of future harvests.

Rice (*Oryza sativa* L.) is one of three major food crops and the main source of nutrients for the global human population; it plays a significant role in facilitating economic development and maintaining national security (Chen et al., 2022). In addition, rice is the first choice to improve and utilize coastal areas and saline-alkali land (Yeo and Flowers, 1986). Despite the prominent increase in the production of rice, we currently face an enormous challenge to enhance the salt tolerance of rice and increase the yield of this crop on salinized agricultural land (Bhatt et al., 2020). Thus, the study of the molecular mechanisms behind salinity resistance is crucial for cultivating new varieties of rice.

Plants have evolved complex and interconnected regulatory networks that enable them to respond and adapt to different environments to withstand salinity stress. For example, sodium (Na^+^) transport and accumulation in plant cells are important for enhancing salt tolerance. This is accomplished by transporters that are responsible for Na^+^ uptake, export and compartmentation. Previous studies demonstrated that the Salt Overly Sensitive (SOS) signaling pathway mediates Na^+^ exclusion (Zhu, 2001). The High affinity K^+^ transporter (HKT)-type transporters are involved in Na^+^ transport (Berthomieu et al., 2003; Hamamoto et al., 2015), and the NHX (Na^+^/H^+^ antiporter)-type transporters facilitate the compartmentation of Na^+^ (Blumwald and Poole, 1985). Studies in Arabidopsis have resulted in significant progress toward understanding the function of SOSs (Quintero et al., 2011; Gong et al., 2020). The SOS functional module is conserved and comprises the SOS1, SOS2, and SOS3 proteins that operate in cereals and possess a high degree of structural conservation between dicots and monocots (Quintero et al., 2002; Martínez-Atienza et al., 2007). In a similar fashion to *AtSOS1*, the *OsSOS1* plasma membrane Na^+^/H^+^ exchanger also plays a substantial role in controlling Na^+^ uptake and root-shoot partitioning to confer higher salt tolerance to rice plants (El Mahi et al., 2019). Members of the NHX family are equally conserved and important for transporting intercellular potassium (K^+^) and maintaining the pH of endomembrane during salt stress (Van Zelm et al., 2020). In Arabidopsis, plants that overexpress *NHX1* display increased tolerance to salt and accumulate Na^+^ in their shoots under conditions of high salinity (Apse et al., 1999). NHXs also transport K^+^ and Na^+^ in crop plants, which has demonstrated relevance for salt tolerance (Almeida et al., 2017). However, the associated haplotypes within these genes remain unknown.

Unlike SOSs and NHXs, Arabidopsis encodes only one HKT gene (*AtHKT1;1*). This contrasts with the multiple copies of HKT-type transporters found in monocot species, such as rice, wheat (*Triticum aestivum* L.) and barley (*Hordeum vulgare* L.) (Hamamoto et al., 2015). In particular, seven functional HKT-type transporters are present in rice, and they encompass two classes (I and II) based on their respective transport activity (Platten et al., 2006). Previous studies reported that rice plants require *OsHKT1;1*, a member of class I, to adapt to salinity stress by reducing the accumulation of Na^+^ in the shoots (Wang et al., 2015). Additionally, a cross between Nona Bokra, a salt tolerant *indica* variety, and Koshihikari, a susceptible *japonica* variety, made it possible to map an essential QTL designated SKC1 (Shoot K^+^ Content) that encodes another member of the HKT family and showed that *OsHKT1;5* mediates the accumulation of K^+^ and Na^+^ in the shoots and xylem sap of rice plants (Ren et al., 2005). Among the HKT gene family, *OsHKT1;5* is an ortholog of *AtHKT1;1*, which primarily detoxifies elevated Na^+^ levels to confer salt tolerance to both Arabidopsis and rice (Garciadeblás et al., 2003). Unlike class I HKT transporters, such as *OsHKT1;1* and *OsHKT1;5*, there is little evidence on the physiological functions of class II genes in plants except for the *OsHKT2;1*-mediated influx of Na^+^ (Horie et al., 2007; Park et al., 2019; Wei et al., 2021a). Additionally, Takagi et al. (2015) used the MutMap method to identify *OsRR22*, a gene responsible for salt tolerance in rice, which allowed the researchers to develop a salt tolerant variety. These data showed that *OsRR22* regulates the transcription of many salt response genes, such as *OsHKT1;1* (Takagi et al., 2015). Although it is well established that HKT proteins play a major role in the salt tolerance of cereal crops, the distinct evolutionary pathways between varieties and the optimal haplotypes remain unknown.

Various transcription factor families, such as MYB, WRKY, and NAC, are also involved in responses to salt stress. In particular, NAC transcription factors are among the largest transcription regulatory gene families in plants, and their members play a vital role in response to abiotic stress (Jeong et al., 2013). Previous studies (Wang et al., 2012b; Yuan et al., 2020) showed that OMTN proteins have characteristics that are typical of NAC transcriptional factors, including the overexpression of *OMTN2*, *OMTN3*, *OMTN4*, and *OMTN6*, which leads to drought sensitivity at the reproductive stage, and changes in the expression of several genes related to stress responses (Fang et al., 2014).

The advent of genome-wide association studies (GWAS) emerged as a powerful strategy to uncover the molecular basis of salt tolerance in rice. GWAS studies can identify more historical recombination, alleles, and wider genetic variation than traditional linkage analysis (Min et al., 2021). With respect to the analysis of genetic variation in different rice accessions, previous studies suggested that wild rice represents an important model for studying salinity resistance, which was preserved by natural selection, and has the potential to improve cultivated rice (Yuan et al., 2020). Using high density SNPs of the rice population to perform GWAS is an available way to identify salt-tolerance genes. Seven genes were identified under salinity stress conditions through GWAS analysis (Liu et al., 2019). Similarly, 295 rice accessions were used to perform GWAS of salt tolerance analysis during the germination stage, and 17 salt-tolerance-related genes were characterized (Yu et al., 2018). Therefore, it is critical to study the extensive genetic diversity of wild rice to identify valuable genes that are involved in salt stress tolerance. Additionally, rice domestication could provide a reliable basis to identify favorable variants for modern breeding (Meyer et al., 2016). In our previous studies, we analyzed the phenotype of 225 rice variety population under normal and low nitrogen conditions and conducted GWAS analysis to obtain four candidate genes (Lv et al., 2021). In this study, we analyzed the salt resistance of 191 rice varieties, performed a GWAS analysis, and combined this information with evaluation scoring results for salt tolerance, genetic differentiation and transcriptomic analysis to identify the genetic underlying basis for salt resistance in rice. Notably, we isolated 275 candidate genes associated with salt tolerance, including the known salt genes *OsHKT1;1* and *SLR1*, which highlighted the reliability and accuracy of our methods. Simultaneously, we conducted haplotype and genetic diversity analyses to explore the predicted salt tolerant genes and the dominant haplotypes to improve rice varieties. The data and genetic resources of this study are highly valuable for correctly characterizing the molecular basis of salt tolerance in rice.

## Materials and Methods

### Plant Materials

The mini-core population used in this study consisted of 184 *Oryza sativa* accessions obtained from the 3,010 Rice Genomes Project (Wang et al., 2018). Seven *Oryza sativa* accessions were provided by Wang et al. (2020). These germplasms originated from 27 different countries (Supplementary Table 1).

### NaCl Treatment and Phenotypic Analysis

Hydroponic experiments were performed at the greenhouse of the China National Rice Research Institute in Hangzhou, China, during the summer of 2021. The grains were germinated in water at 37°C in the dark for 3 days. We examined the effects of salt stress in rice at the seedling stage and set different levels of NaCl concentration (50 mM NaCl, 100 mM NaCl, 200 mM NaCl) for pre-experiments. Descriptive statistics of the phenotypes related to salt tolerance at the seedling stage of 191 rice accessions are presented in Supplementary Table 2. A wide range of phenotype values were observed under 100 mM NaCl solution in the salt traits evaluation, which resulted in the most diverse phenotypic distribution and facilitated discrimination of accessions with different salt tolerance levels. The seedlings were cultured in a nutrient solution (pH 5.5–5.8) with the following composition (Peethambaran et al., 2018): 1.425 NH_4_NO_3_, 0.42 NaH_2_PO_4_, 0.510 K_2_SO_4_, 0.998 CaCl_2_, 1.643 MgSO_4_, 0.168 Na_2_SiO_3_, 0.125 Fe-EDTA, 0.019 H_3_BO_3_, 0.009 MnCl_2_, 0.155 CuSO_4_, 0.152 ZnSO_4_, and 0.075 Na_2_MoO_4_. The solutions were changed every 2 days. After 2 weeks of hydroponic culture, 48 plants per line of the 14-day-old seedlings with uniform growth were exposed to a 100 mM solution of NaCl as the salt stress treatment, while the remaining 48 plants per line with uniform growth were cultured in a normal solution for an additional 14 days as the control (Jahan et al., 2020, 2021). The whole experiment was conducted in a randomized complete block design with two biological replications, and all of the tested samples were efficient.

Ten seedlings of each accession were evaluated for salt tolerance grades, and five salt tolerance indices (1, 3, 5, 7, and 9) were divided based on plant growth and development phenotypes after salt treatment (Gregorio et al., 1997). This scoring discriminated the susceptible from the tolerant and moderately tolerant genotypes. Plants with lower salt tolerance scores displayed visual symptoms that manifested as brown leaf tips, yellowing leaves, dry leaves, reduced shoot growth, and stunted height. These phenotypes increased with the time of stress.

### Genome-Wide Association Study

The sequence data rice accessions used for GWAS were obtained from the 3,000 Rice Genomes Project (Wang et al., 2018). The SNP data were filtered with the following criteria: minor allele frequency (MAF) >0.05 (Visscher et al., 2012) and missing rate ≤30% (Yu et al., 2021). The linkage disequilibrium (LD) parameter r^2^ between pairwise SNPs was calculated using PopLDdecay. The physical LD decay distance was estimated as the position where r^2^ dropped to half of its maximum value. We estimated an LD decay distance of 86.8 kb. The efficient mixed model analysis feature of the EMMA eXpedited (EMMAX) software was utilized for GWAS analysis. The significance threshold was calculated using the formula “−log10 (1/the effective number of independent SNPs).” The threshold was set at −log *P* = 5 to identify significant association signals (Gao et al., 2020; Lv et al., 2021), and the candidate genes were detected as those within 200 kb of the significant association signals (Yu et al., 2017) using a mixed linear model (MLM) model (Kang et al., 2010). Plots that represented the GWAS results (Manhattan and Quantile-Quantile plots) were generated using the package qqman in R 3.4.2 (Turner, 2014). Annotations were added to the filtered VCF files using SnpEff software, and missense variant sites were selected for haplotype analysis of the target gene as the result.

### Data Samples and Single Nucleotide Polymorphism Calling for Genetic Differentiation

A total of 159 *indica*, 146 *temperate japonica*, and 41 *tropical japonica* varieties, as well as 53 *Oryza rufipogon*, 163 *Oryza glaberrima*, and 83 *Oryza barthii* samples, were obtained from previous studies (Huang et al., 2012b; Cubry et al., 2018; Wang et al., 2018). The raw Illumina short reads were filtered using Trimmomatic 0.36 (Bolger et al., 2014). The clean reads were mapped to the Nipponbare reference genome (MSUv7) (Kawahara et al., 2013) using BOWTIE2 v2.2.1 (Langmead and Salzberg, 2012) with default parameters. SAMtools v1.8 was used for SNP calling, and the SNPs were filtered using VCFtools (Danecek et al., 2021) with the following parameters: “–maf 0.001 –max-missing 0.9 –remove-indels –min-alleles 2 –max-alleles 2.”

### Population Differentiation Statistics

The population differentiation statistics (*F*_*ST*_) between wild and *indica*, wild and *japonica*, and *O. glaberrima* and *O. barthii* were estimated separately for each window using VCFtools (Danecek et al., 2011). *F*_*ST*_ was calculated with the following parameters: “–fst-window-size 100,000 –fst-window-step 10,000.” Sliding windows with the top 5% of *F*_*ST*_ values were identified as divergent windows. Genes that overlapped the *F*_*ST*_ divergent windows were annotated based on the MSUv7 annotation (Kawahara et al., 2013).

### RNA-Seq

The cultivars 93-11 and PA64s, as well as their salt tolerant plants (based on phenotyping results), were selected for RNA-Seq analysis (Accession: PRJNA831421)^1^. Total RNA was extracted with TRIzol, and the RNA-Seq libraries were prepared using two biological replicates for each species. After filtering out non-conforming sequences with Trimmomatic (0.36) (Bolger et al., 2014), TopHat2 (v2.0.12) (Trapnell et al., 2012) was used to align the cleaned data to the reference genome (Kawahara et al., 2013). The default parameters in the Cufflinks (v2.2.1) were used to obtain the gene expression level (Fragments per kilobase per million, FPKM) of each gene (Trapnell et al., 2012). Meanwhile, genes with *P*-value < 0.05, *Q*-value < 0.05 and FPKM difference greater than 1.5 times between the control group and the treatment group obtained by Cuffdiff (v2.2.1) were regarded as differentially expressed genes (DEGs). TBtools (v1.098723) (Chen et al., 2020) was applied to map the DEGs of the GO database^2^, and it was also be used to calculate the number of genes in each term to obtain a list of genes with a certain GO function and the statistics of the number of genes. The differential genes of GO item species with significantly enriched differential genes and *P*-value < 0.05 will be retained for analysis.

## Results

### Geographic Distribution and Population Structure

**FIGURE 1:**
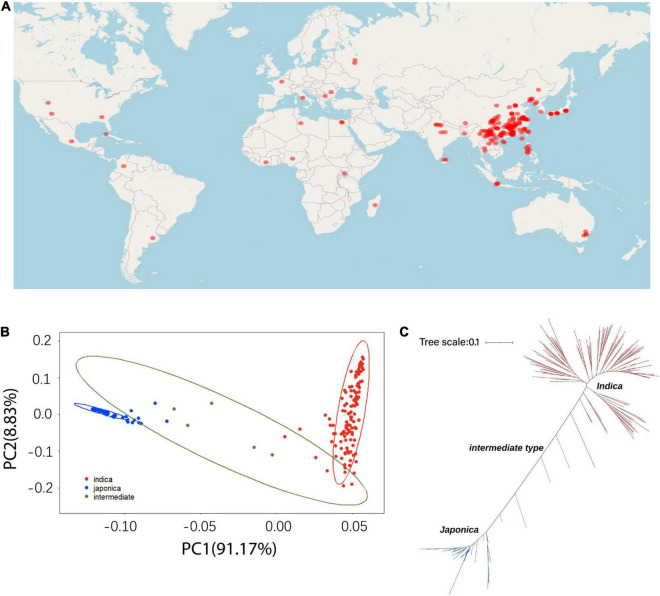
The geographic distribution and population genetic structure across the 191 rice cultivated accessions. **(A)** Geographic distribution. **(B)** Principal component analysis. **(C)** Neighbor-joining tree of the 191 cultivated rice accessions.

The different accessions used in this study are shown in Figure 1. A total of 191 *O. sativa* accessions were collected from 27 different regions of the world (Figure 1A), which represents a large variation of geographical origins and genetic diversity for the accessions of cultivated rice. A principal component analysis (PCA) was performed with the high-quality SNPs/indels to mine the population structure in the rice accessions. Clear subpopulation structures were observed, which resulted in three subpopulations designated *indica* and *japonica* with the admixture accessions located between the two groups (Figure 1B). The resulting neighbor-joining tree showed consistency with the PCA analysis and identified 129 *indica*, 57 *japonica*, and five intermediate type accessions, which largely supported the classification of the 191 accessions (Figure 1C). There were five intermediate accessions, which could have resulted from occasional historical hybrids between *indica* and *japonica* that experienced partial reproductive isolation.

### Evaluation of Salt Tolerance Among the Accessions

**FIGURE 2:**
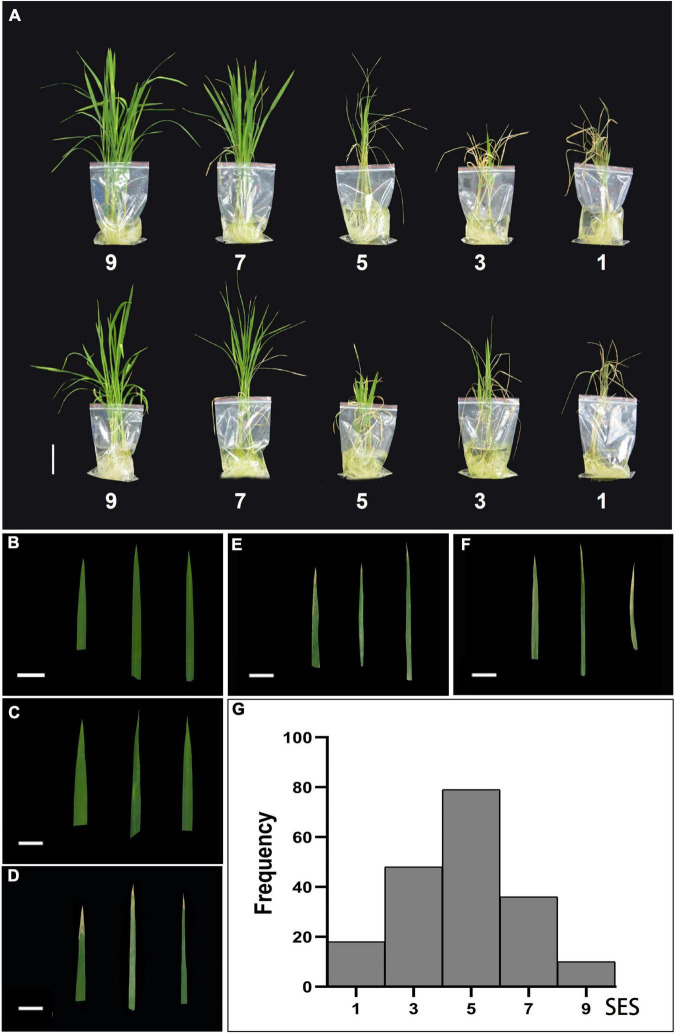
Classification and frequency histogram of the salt tolerance evolution score (SES) in the entire population. **(A)** Morphological responses of ten different rice varieties under 100 mM NaCl stress based on the salt tolerance evolution score (SES). Scale bar: 5 cm. Phenotypic characterization of five SES [9 **(B)**, 7 **(C)**, 5 **(D)**, 3 **(E)**, 1 **(F)**] of the visual symptoms in leaves. Scale bar: 2 cm. **(G)** Frequency histogram of the SES in the entire population.

We examined the effects of salt stress in rice at the seedling stage and established different levels of NaCl concentrations for pre-experiments. A wide range of phenotypic values was observed when the accessions were treated with a 100 mM solution of NaCl to evaluate the salt traits. This resulted in the most diverse phenotypic distribution and facilitated the discrimination of accessions with different levels of salt tolerance. We treated 14-day-old seedlings from the 191 rice cultivated accessions with control (0 mM^–1^) and high NaCl/stress (100 mM^–1^) solutions. The plants displayed distinct salt tolerance variation across these populations that were analyzed. We then evaluated salt tolerance based on plant growth and development phenotypes. The evaluation of salt tolerance scores was divided into five grades (1, 3, 5, 7, and 9) using a modified standard evaluating score (Table 1) based on the visual symptoms of salt toxicity. This evaluation score distinguishes susceptible from tolerant and moderately tolerant phenotypes (Figure 2). We found no significant differences in plant growth and development between these accessions, and all the plants, such as Qiuqianbai (NS47) and Hongainuo (NS112), displayed a high SES under both normal and high salt stress conditions. We classified the accessions with a score of being highly tolerant to salt. Compared with normal conditions, the low SES accessions, particularly Heibiao (NS1) and Vietnam Zaodao (NS6), grew and developed poorly. In fact, almost all the plants died or were severely damaged under high salt stress conditions, and these accessions appear to be highly susceptible to salt stress. There was also a clear distinction between visual symptoms among the tolerant, moderate, and susceptible plants. The mean, standard deviation (SD), and coefficient of variation values for the SES are shown in Supplementary Table 2. This indicates the 191 cultivated rice accessions in the GWAS panel exhibited considerable natural variation in their degree of salt tolerance and had a very high level of genetic diversity.

**TABLE 1:** The standard evaluation score (SES) salt-tolerance.

Score	Observation	Tolerance
1.	Almost all plants dead or dying	Highly susceptible
3.	Complete cessation of growth, most leaves dry, some plants dying	Susceptible
5.	Growth severely retarded, most leaves rolled, only a few are elongating	Moderately tolerant
7.	Nearly normal growth, but leaf tips or few leaves whitish and rolled	Tolerant
9.	Normal growth, no leaf symptoms	Highly tolerant

*The standard evaluation score (SES) was modified from IRRI Gregorio et al. (1997).*

### Genome-Wide Association Study and Candidate Genes for Salt Tolerance

**FIGURE 3:**
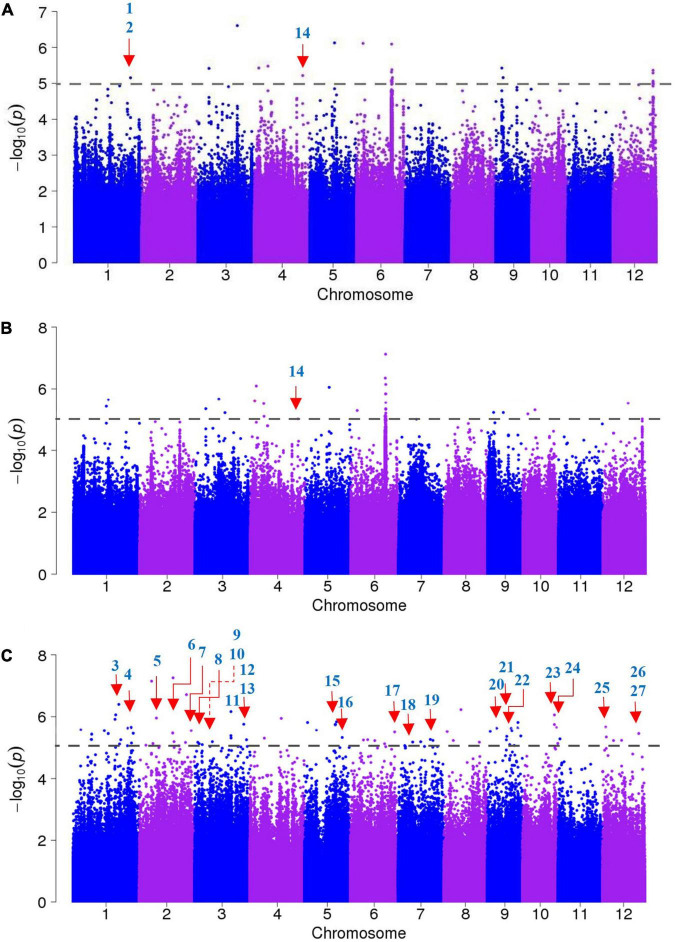
Genome-wide association studies of salt tolerance evolution scores (SES). **(A)** Manhattan plots of the entire accessions panel, **(B)** the *indica* accessions panel, **(C)** the *japonica* accessions panel. The known candidate genes responsible for salt sensitivity are indicated with red arrows, the numbers correspond to the genes presented in Table 2.

A GWAS was performed to identify candidate genes based on ∼3.82 million SNPs, with missing rates ≤30% and MAF >0.05. These polymorphisms covered the whole rice genome, and we used MLM to calculate associations. In particular, we established a threshold of −log *P* = 5 as a significant association standard. Overall, we identified 155 SNPs associated with salt tolerance, including 24 that were found across the entire panel, 37 in the *indica* panel and 94 in the *japonica* panel (Figure 3).

**FIGURE 4:**
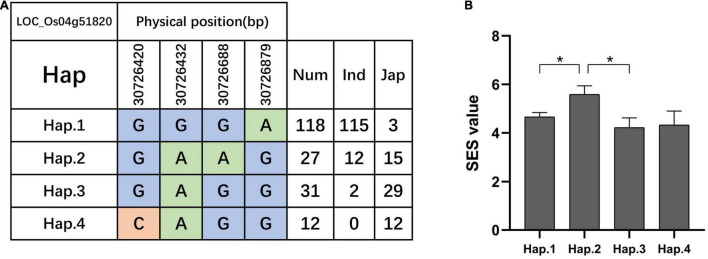
Haplotype analyses of *OsHKT1;1* (*LOC_Os04g51820*). Gene structure **(A)** and SES **(B)** of different haplotypes of *OsHKT1;1.* T-test, *P < 0.05.

We searched for candidate genes within the genomic intervals of significant SNPs using the Rice Genome Annotation Project^3^ and published research on salt tolerance as references. We found 275 candidate genes in total (Supplementary Tables 3–5), including 27 genes that had been previously associated with salt sensitivity (Table 2). In the entire GWAS panel, we found 74 candidate genes in the intervals that corresponded to the 24 significant SNPs. We identified two candidate genes, *ZFP179* and *OsDSR2*, in the SNP peak Chr1_36063662 that were associated with salt tolerance and located at 61.6 kb and 58.6 kb, respectively. Additionally, *OsHKT1;1* (*LOC_Os04g51820*) was co-detected in the entire GWAS and *indica* panels. This gene is a member of the HKT family and plays a significant role in preventing sodium toxicity in leaf blades by reducing the accumulation of Na^+^ in shoots under conditions of high salinity. At the transcription level, *OsHKT1;1* is activated by the *OsMYBc* transcription factor. We further studied the role of *OsHKT1;1* by conducting haplotype analyses on the 191 rice varieties to identify elite haplotypes using all non-synonymous SNPs within their open reading frames (ORFs; Figure 4). We identified four distinct haplotypes based on four SNPs, which were responsible for the genetic differences observed between *indica* and *japonica*. Hap.1 was solely present in *indica*; Hap.2 was present in both *indica* and *japonica*, and Hap.3 and Hap.4 were predominantly present in *japonica*. Significant differences in salt tolerance indices were detected between Hap.2 and Hap.3. Moreover, the accessions that harbored the Hap.2 genotype displayed a higher SES than the other accessions, particularly Haobayong1 (NS153) and Menjiading (NS154). This indicates that Hap.2 confers salt tolerance to seedlings. These results further validate the role played by *OsHKT1;1* in the regulation of variation in salt tolerance at the seedling stage.

**TABLE 2:** Summary of known candidate genes for salt sensitivity.

No.	Gene ID	Gene name	Description	References
1.	LOC_Os01g62190	ZFP179	Cys2/His2-type zinc finger protein	Sun et al., 2010
2.	LOC_Os01g62200	OsDSR2	DUF966-stress repressive gene 2	Luo et al., 2014
3.	LOC_Os01g48800	OsPUP4	Purine permease putative expressed	Yin et al., 2020
4.	LOC_Os01g65500	OsCLC1	Chloride channel protein putative expressed	Diedhiou and Golldack, 2006
5.	LOC_Os02g18930	OsCBL8	Calcineurin B putative expressed	Ma et al., 2010
6.	LOC_Os02g43010	OsVPE3	Vacuolar-processing enzyme precursor putative expressed	Lu et al., 2016
7.	LOC_Os02g50350	OsDHODH1	Dihydroorotate dehydrogenase protein putative expressed	Liu et al., 2009
8.	LOC_Os03g06370	OsNBL3	PPR repeat domain containing protein putative expressed	Qiu et al., 2021
9.	LOC_Os03g20680	OsLEA3-2	Late embryogenesis abundant protein 1 putative expressed	Duan and Cai, 2012
10.	LOC_Os03g20790	MHZ6	Ethylene-insensitive 3 putative expressed	Yang et al., 2015
11.	LOC_Os03g49990	SLR1	GRAS family transcription factor domain containing protein expressed	Mo et al., 2020
12.	LOC_Os03g60430	OsIDS1	AP2 domain containing protein expressed	Julkowska, 2018
13.	LOC_Os03g60560	ZFP182	ZOS3-21 – C2H2 zinc finger protein expressed	Huang et al., 2012a,b
14.	LOC_Os04g51820	OsHKT1;1	High-Affinity Potassium Transporter	Wang et al., 2015
15.	LOC_Os05g35410	*OsAKT2*	Potassium channel AKT2 3 putative expressed	Tian et al., 2021
16.	LOC_Os05g50710	*OsLEA5*	Late embryogenesis abundant protein putative expressed	He et al., 2012
17.	LOC_Os06g47860	*OsSIDP366*	Expressed protein	Guo et al., 2016
18.	LOC_Os07g08860	*OsSIK2*	S-domain receptor-like protein kinase putative expressed	Chen et al., 2013
19.	LOC_Os07g37400	*OsMsr9*	OsFBX257 – F-box domain containing protein expressed	Xu et al., 2014
20.	LOC_Os09g26400	*OsDSG1*	Zinc finger C3HC4 type domain containing protein expressed	Park et al., 2010
21.	LOC_Os09g26780	*OsJAZ8*	Zinc-finger protein putative expressed	Peethambaran et al., 2018
22.	LOC_Os09g37949	*OsRPK1*	Serine threonine-protein kinase SRPK1 putative expressed	Cheng et al., 2009
23.	LOC_Os10g38950	*OsMPK6*	CGMC_MAPKCMGC_2_ERK.14 – CGMC includes CDA MAPK GSK3 and CLKC kinases expressed	Wang et al., 2014
24	LOC_Os10g41400	*OsMSRA4.1*	Peptide methionine sulfoxide reductase putative expressed	Guo et al., 2009
25	LOC_Os12g05440	*CYP94C2b*	Cytochrome P450 putative expressed	Kurotani et al., 2015
26	LOC_Os12g38180	*OsHSP23.7*	Heat shock cognate 70 kDa protein 2 putative expressed	Zou et al., 2012
27	LOC_Os12g38400	*OsMYB91*	MYB family transcription factor putative expressed	Zhu et al., 2015

Among the remaining 24 candidate genes, *OsPUP4*, *OsNBL3*, *MHZ6*, *OsIDS1*, and *OsJAZ8* were associated with salt tolerance. Additionally, 19 genes, including *OsCLC1*, *OsCBL8*, *OsVPE3*, *OsDHODH1*, *OsLEA3-2*, *SLR1*, *ZFP182*, *OsAKT2*, *OsLEA5*, *OsSIDP366*, *OsSIK2*, *OsMsr9*, *OsDSG1*, *OsRPK1*, *OsMPK6*, *OsMSRA4.1*, *CYP94C2b*, *OsHSP23.7*, and *OsMYB91*, were identified as salt sensitivity genes.

### Genetic Differentiation of Candidate Genes Between Different Subpopulations

**FIGURE 5:**
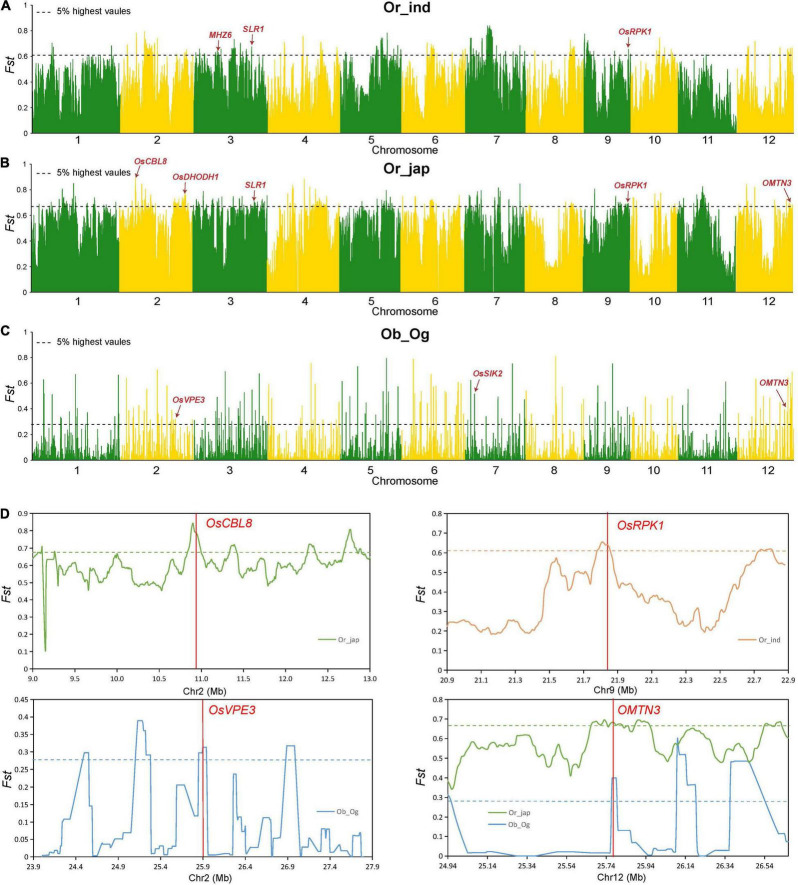
Genomic differentiation statistics. **(A–C)** Genomic differentiation between *O. rufipogon* and *indica* (ind) **(A)**; *O. rufipogon* (Or) and *japonica* (jap) **(B)**; and *O. barthii* (Ob) and *O. glaberrima* (Og) **(C)**. Red arrows indicate the divergent regions overlapping with reported salt tolerance genes. **(D)** Genetic differentiation of several important genes. Green, orange, and blue lines represent the *F*_*ST*_ between *O. rufipogon* and *indica*, *O. rufipogon* and *japonica*, and *O. barthii* and *O. glaberrima*, respectively. Horizontal dashed lines correspond to the top 5% threshold. Red vertical lines represent the location of the reported genes.

To examine the genetic basis behind the differences in salt tolerance observed among subpopulations at the seedling stage, we assessed 159 *indica* (ind), 146 temperate *japonica* and 41 tropical *japonica* (jap), as well as 53 wild *O. rufipogon* (Or), 163 *O. glaberrima* (Og) and 83 *O. barthii* (Ob) samples. We performed an *F*_*ST*_ analysis (Figure 5) and haplotype analysis (Supplementary Figure 1) separately, while the combined analysis was performed using the significant differentiated regions and the candidate genes as obtained by GWAS. We observed seven candidate genes in the domestication region of Asian and African rice, namely *LOC_Os02g21810*, *LOC_Os05g35170* (*IDEF2*), *LOC_Os10g41130*, *LOC_Os10g41200* (*MYBS3*), *LOC_Os10g41260*, *LOC_Os10g41330*, and *LOC_Os12g41680* (*OMTN3*). These genes could have been selected simultaneously during the domestication of Asian and African rice varieties (Supplementary Table 6). Additionally, among the 27 genes previously associated with salt sensitivity, *OsVPE3* (*LOC_Os02g43010*) was observed in the domestication region between the Ob and Og subpopulations; *MHZ6* (*LOC_Os03g20790*) was found in the domestication region between the Or and *indica* subpopulations, and *OsCBL8* (*LOC_Os02g18930*), *OsDHODH1* (*LOC_Os02g50350*), *SLR1* (*LOC_Os03g49990*), *OsRPK1* (*LOC_Os09g37949*) were identified in the domestication region between the Or and *japonica* subpopulations. These results suggest that these candidate genes reside in selective sweep regions and could act as targets for the molecular improvement of rice salt tolerance.

### Differentially Expressed Rice Genes From RNA-Seq Data in Response to Salt Stress

A transcriptome analysis is an effective strategy to identify the genes associated with a particular trait. To investigate transcriptional changes in rice under salinity stress, we selected two varieties of plants from the 191 accessions panel, specifically 93-11 and PA64s. These plants exhibited significant phenotypic differences under 0 mM (93-11_*ST0;*_ PA64s_*ST0*_) and 100 mM (93-11_*ST100*_; PA64s_*ST100*_) NaCl conditions and were used for further RNA-Seq analysis. Clearly observable symptoms of salt injury were apparent in the 93-11_*ST100*_ seedlings after treatment with NaCl. The visual symptoms consisted of leaves that were mostly dried, the complete cessation of plant growth, and death of the plants. These effects were exacerbated by the amount of time under stress. We found similar but milder symptoms in PA64s_*ST100*_.

To evaluate the potential molecular mechanisms that underlie salt tolerance, we combined the 93-11 and PA64s cultivars with their corresponding salt tolerant treatment plants and screened the DEGs of the two salt-tolerant control groups. A total of 2,821 and 1,249 DEGs that corresponded to two salt-stress pairwise comparisons were identified in ST1 (93-11_*ST0*_ vs. 93-11_*ST100*_) and ST2 (PA64s_*ST0*_ vs. PA64s_*ST100*_), respectively. We also noted that a higher number of DEGs were identified in ST1 (1,452 upregulated and 1,368 downregulated) than ST2 (843 upregulated and 406 downregulated).

**FIGURE 6:**
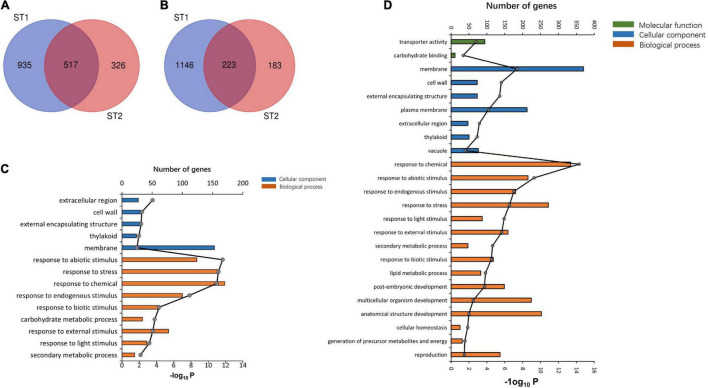
The transcriptome analysis of salt stress between 93 and 11 and PA64s. Venn diagrams showing the common **(A)** upregulated and **(B)** downregulated DEGs among ST1 (93-11_*ST0*_ vs. 93-11_*ST100*_) and ST2 (PA64s_*ST0*_ vs. PA64s_*ST100*_). GO classification at **(C)** ST1 and **(D)** ST2.

The GO enrichment analysis indicated that the upregulated DEGs in ST1 and ST2 were highly enriched in the GO terms “response to chemical” and “stress.” The DEGs in ST1 that were enriched in the GO terms related to “membrane process” and “plasma membrane” were downregulated, while the genes enriched in “response to stress” and “membrane” were downregulated in ST2 (Supplementary Tables 7, 8). There was a total of 517 co-upregulated and 223 co-downregulated genes in ST1 and ST2 (Figure 6).

The DEGs investigated by RNA-Seq provide important clues to identify potential candidate genes responsible for rice growth and development under salt stress. A total of 30 candidate genes associated with salt tolerance were obtained by integrating GWAS and transcriptomic DEGs analyses. These cloned genes included 13 with no known association with salt tolerance and 17 candidate genes whose functions were unknown. Using the previously reported genes and published research on salt sensitivity as references, we found *LOC_Os12g41680* (*OMTN3*) to be associated with the response to endogenous stimulus process on the candidate interval Chr12_25666325. This gene encodes the no apical meristem protein. Interestingly, *OMTN3* was a DEG in ST2 and upregulated in PA64s under salt stress. Simultaneously, previous studies indicated that the OMTN genes were responsive to abiotic stresses, showed diverse spatiotemporal patterns of expression in rice and regulated numerous stress response genes; in particular, development and metabolism were altered in plants that overexpressed *OMTN3*. The candidate gene *LOC_Os10g41260* encodes a MYB family transcription factor and was detected in the candidate interval Chr10_22142925. The gene was significantly enriched for GO biological processes related to chemical and endogenous stimulus response. In addition, *LOC_Os12g41680* and *LOC_Os10g41260* were selected during the diversification of the Or and *japonica* subspecies and the Ob and Og subspecies. We further performed haplotype analysis on the candidate gene *LOC_Os12g41680* and *LOC_Os10g41260* on 159 *indica* (ind), 146 *temperate japonica* (Tej), 41 *tropical japonica* (Trj), and 53 *O. rufipogon* (Or) to investigated different haplotypes under selection during domestication. *LOC_Os10g41260* was classified into two haplotypes, which Hap.1 only distributed in cultivated rice (mainly in *temperate japonica*) and Hap.2 mainly distributed in *indica* varieties (Supplementary Figure 1A). For the candidate gene *LOC_Os12g41680* haplotype analysis, we identified three types that Hap.1 mainly distributed in *japonica* subspecies, Hap.2 mainly distributed in *indica* and Hap.3 mainly distributed in wild rice (Or) (Supplementary Figure 1B). The haplotype analysis showed consistency with the *F*st analysis, suggested that there are different haplotypes emerged during the domestication between the different subpopulations. Based on the evaluation scores for salt tolerance, GWAS, genetic differentiation and transcriptome analysis, *LOC_Os12g41680* and *LOC_Os10g41260* can be used as candidate genes for salt tolerance in rice.

## Discussion

The growth and development of rice are inhibited under salt stress, and rice varieties were required to rapidly adapt to high salinity environments and grow well under salt stress over the course of evolution (Yamaguchi and Blumwald, 2005). To date, despite previous research that focused on the molecular mechanisms behind salt tolerance in rice, study of the adaptive mechanisms that integrate various pathways and molecular components remains challenging (Flowers and Flowers, 2005). Over the past few decades, several genes and QTLs associated with different traits for salt tolerance have been identified. However, only *SalTol*/*SKC1* has been utilized in rice breeding (Thomson et al., 2010; Wang et al., 2015). There is a critical current challenge to enhance the tolerance to salt in rice to increase yields on salinized agricultural land (Ponce et al., 2021). Accordingly, the detection and mapping of genes and QTLs is crucial to identify new salt tolerance genes in the rice seedling stages.

Unraveling the genetic factors for salt tolerance in plants is a challenging endeavor. Association mapping emerged as a useful tool to identify alleles and QTLs associated with agronomically important traits (Thomson et al., 2010; Lv et al., 2021; Wei et al., 2021b). The collection of a wide range of germplasm resources with different genetic backgrounds is an essential step in association analysis. In this study, we collected a set of mini-core populations from different regions around the world, primarily from the 3,000 Rice Genomes Project (Fang et al., 2014). However, Wang et al. (2018) also provided seven accessions. Controlling for population structure has considerable implications for GWAS analysis, and population stratification can either introduce or remove spurious associations between genotypes and phenotypes (Stich et al., 2006). The population analyzed consisted of 191 rice genotypes that originated from 27 countries, corresponding to a sample with sufficient genetic variation for feasible association analysis to be conducted with the ultimate goal of discovering beneficial candidate genes that improve salt tolerance in rice. An ideal germplasm resource population should contain rich genotypic and phenotypic data (Pace et al., 2015; Valdisser et al., 2020). The data shown here consist of genotypic data for ∼3.82 million SNP markers from 191 germplasms, with the phenotypic variation used to conduct a GWAS analysis for salt tolerance. We considered the salt tolerance evaluation score to be a reliable measure to evaluate salt tolerance with phenotypic variation.

We identified 275 candidate genes within the candidate intervals. These genes were detected using the Rice Genome Annotation Project and published research on salt tolerance as references. Among these genes, there were 27 that had been previously identified as related to salt sensitivity, including five salt tolerant and 22 salt sensitivity genes. Surprisingly, *OsHKT1;1* was identified as a candidate gene in our association analysis. This gene is located in the candidate interval chr4_30893016, and previous studies reported that this member of the HKT family plays an important role in reducing the accumulation of Na^+^ in shoots to circumvent salt stress (Platten et al., 2006). Sodium transporters mediate Na^+^-specific transport or Na^+^-K^+^ co-transport and are known to play key roles in plant tolerance to salt stress, particularly in HKT transporter-dependent fashion (Ren et al., 2005; Wang et al., 2012a). Association analysis enables the evaluation of a large number of alleles in different populations (Krill et al., 2010). To further explore allelic differences, we performed haplotype analysis on the candidate gene *OsHKT1;1* and identified four distinct haplotypes based on four SNPs that differentiated the *indica* and *japonica* varieties. Accessions that harbored the Hap.2 genotype displayed a higher SES than those that harbored other haplotypes, particularly Haobayong1 and Menjiading. This indicates that salt tolerance is present in seedlings. Elite Hap.2 alleles of *OsHKT1;1* was studied and will serve as potential candidates to genetically improve salt tolerant rice.

Salt tolerance diverged during the domestication of cultivated rice and enabled the plants to adapt to changing ecological habitats. To clarify the genetic basis of differences in salt tolerance among subgroups, we assessed African and Asian wild and cultivated rice by separately performing an *F*_*ST*_ analysis. The combined analysis was conducted using the significantly differentiated sites, and the candidate genes were obtained using a GWAS analysis. We found that seven genes were simultaneously selected during the domestication of Asian and African rice, namely *OsVPE3* (*LOC_Os02g43010*), *MHZ6* (*LOC_Os03g20790*), *OsCBL8* (*LOC_Os02g18930*), *OsDHODH1* (*LOC_Os02g50350*), *OsSIK2* (*LOC_Os07g08860*), *SLR1* (*LOC_Os03g49990*), and *OsRPK1* (*LOC_Os09g37949*). The genes for salt tolerance that were identified can be differentiated and applied at the seedling stage to provide important information for the identification and pyramid breeding of salt tolerance genes in rice plants.

In addition to performing a GWAS analysis on salt tolerance across 191 rice cultivars, we combined RNA-Seq data of a susceptible *indica* cultivar (93-11) and a salt tolerant *japonica* cultivar (PA64s) to identify the genetic loci that confers salt resistance in rice. By integrating GWAS and transcriptomic analyses, 30 genes from both DEGs and GWAS candidate genes were identified. Among these genes, *OMTN3* (*LOC_Os12g41680*) is a NAC transcription factor (Jeong et al., 2013), a family of genes that are widely distributed in plant species. For example, the *OsNAC6* gene is one of the many NAC genes in rice that are associated with cold, salt, drought and abscisic acid (ABA) responses (Nakashima et al., 2007). In addition, the overexpression of another gene NAC transcription factor, *SNAC1*, significantly improves drought and salt tolerance in rice and regulates the expression of many stress-related genes (Redillas et al., 2012). *OMTN3* is a DEG that was identified in the PA64s test group and is upregulated under salt stress in these plants. Moreover, it has been previously reported that *OMTN3* negatively regulates drought tolerance in rice. Combining annotation and metabolic function information enabled the initial prediction that *OMTN3* was a candidate gene associated with salt tolerance. Furthermore, we also focused on another candidate gene, *LOC_Os10g41260*, which encodes a MYB family transcription factor. *LOC_Os10g41260* was significantly enriched for GO biological processes related to the chemical and endogenous stimulus responses. Interestingly, *LOC_Os12g41680* and *LOC_Os10g41260* were selected simultaneously during the domestication of Asian and African rice, that the results of haplotype analysis showed consistency with the *F*st analysis. The salt tolerance evaluation scores, GWAS, genetic differentiation and transcriptome analysis led us to propose that the genes *LOC_Os12g41680* and *LOC_Os10g41260* can be used as candidate genes to affect the regulation of rice salt tolerance, even though further molecular functional verification needs to be conducted.

Overall, our study provides a theoretical basis to select and breed salt tolerant rice varieties. In particular, enhanced knowledge on the genetic information behind the complex mechanisms associated with this trait in rice will help to facilitate this endeavor.

## Data Availability Statement

The datasets presented in this study can be found in online repositories. The names of the repository/repositories and accession number(s) can be found below: https://www.ncbi.nlm.nih.gov/, PRJNA831421.

## Author Contributions

LG, LS, and QQ designed the experiment. JM, YW, and NJ performed all the phenotype evaluations. YL, FX, and MH performed analysis and interpretation of the data. YL and HW drafted the manuscript. All authors have read and agreed to the published version of the manuscript.

## Conflict of Interest

The authors declare that the research was conducted in the absence of any commercial or financial relationships that could be construed as a potential conflict of interest.

## Publisher’s Note

All claims expressed in this article are solely those of the authors and do not necessarily represent those of their affiliated organizations, or those of the publisher, the editors and the reviewers. Any product that may be evaluated in this article, or claim that may be made by its manufacturer, is not guaranteed or endorsed by the publisher.

## Footnotes

1
https://www.ncbi.nlm.nih.gov/

2
http://www.geneontology.org/

3
https://www.ricedata.cn/
