# Supplementary material for: Combining GWAS, Genome-Wide Domestication and a Transcriptomic Analysis Reveals the Loci and Natural Alleles of Salt Tolerance in Rice (Oryza sativa L.)
Source: Front Plant Sci. 2022 Jun 16;13:912637. doi: 10.3389/fpls.2022.912637 (PMC9248812; doi:10.3389/fpls.2022.912637)
Supplement: Supplementary file 1 [file Data_Sheet_1.PDF]

# *Supplementary Material*

**Supplementary Figure S1|** Haplotype analyses of *LOC_Os10g41260* (A) and *LOC_Os12g41680* (B) on *indica* (ind), *temperate japonica* (Tej), *tropical japonica* (Trj), and *O. rufipogon* (Or). Num, number.

A

| LOC_Os10g41260 | Physical position(bp) | | | | | | | | | | | | | | | | | | |
|---|---|---|---|---|---|---|---|---|---|---|---|---|---|---|---|---|---|---|---|
| Hap | 22105798 | 22105348 | 22105384 | 22105464 | 22105555 | 22105567 | 22105798 | 22105873 | 22106230 | 22106748 | 22106817 | 22106947 | 22106948 | 22107127 | Num. | Ind | Trj | Tej | Or |
| Hap.1 | C | C | C | G | G | T | C | T | G | G | C | T | C | T | 187 | 24 | 34 | 129 | 0 |
| Hap.2 | T | T | T | A | A | C | T | C | A | A | A | C | T | G | 77 | 74 | 0 | 0 | 3 |

B

| LOC_Os12g41680 | Physical position(bp) | | | | | | | | | |
|---|---|---|---|---|---|---|---|---|---|---|
| Hap | 25765362 | 25766089 | 25766508 | 25770516 | 25770558 | Num. | Ind | Trj | Tej | Or |
| Hap.1 | G | A | A | C | G | 186 | 8 | 39 | 139 | 0 |
| Hap.2 | A | T | G | T | A | 129 | 125 | 0 | 1 | 3 |
| Hap.3 | A | A | G | C | G | 19 | 19 | 0 | 0 | 35 |

**Supplementary Table S1|** Summary of 191 rice natural varieties, SES, subpopulations and origin.

| Num. | SES | Subpopulation | Ori. |
|---|---|---|---|
| BN1 | 3 | japonica | China |
| BN2 | 5 | japonica | China |
| BN3 | 5 | japonica | China |
| BN4 | 5 | japonica | Japan |
| BN5 | 5 | japonica | Vietnam |
| BN6 | 3 | indica | Vietnam |
| BN7 | 1 | indica | India |
| BN8 | 5 | indica | India |
| BN9 | 5 | indica | Sri lanka |
| BN10 | 5 | japonica | Soviet Union |
| BN11 | 5 | indica | Romania |
| BN12 | 5 | japonica | Bulgaria |

| | | | |
|---|---|---|---|
| BN13 | 7 | indica | Mexico |
| BN14 | 3 | indica | Australia |
| BN15 | 7 | indica | Thailand |
| BN16 | 7 | japonica | Indonesia |
| BN17 | 5 | indica | Phillipines |
| BN18 | 5 | indica | Phillipines |
| BN19 | 5 | indica | India |
| BN20 | 5 | indica | India |
| BN21 | 5 | indica | Sri lanka |
| BN22 | 3 | japonica | Argentina |
| BN23 | 3 | japonica | Egypt |
| BN24 | 5 | indica | Ivory Coast |
| BN25 | 7 | indica | Uganda |
| BN26 | 5 | japonica | Japan |
| BN27 | 5 | japonica | Japan |
| BN28 | 7 | japonica | Japan |
| BN29 | 7 | japonica | Japan |
| BN30 | 3 | indica | Indonesia |
| BN31 | 5 | indica | Egypt |
| BN32 | 5 | indica | Madagascar |
| BN33 | 3 | japonica | Australia |
| BN34 | 3 | japonica | Australia |
| BN35 | 3 | japonica | Australia |
| BN36 | 5 | indica | China |
| BN37 | 5 | indica | China |
| BN38 | 3 | indica | China |
| BN39 | 7 | indica | China |
| BN40 | 5 | indica | China |
| BN41 | 5 | indica | China |
| BN42 | 5 | indica | China |
| BN43 | 5 | indica | China |
| BN44 | 7 | japonica | China |
| BN45 | 5 | japonica | China |
| BN46 | 5 | japonica | China |
| BN47 | 9 | indica | China |
| BN48 | 5 | indica | China |
| BN49 | 3 | indica | China |
| BN50 | 5 | indica | China |
| BN51 | 7 | indica | China |
| BN52 | 5 | indica | China |
| BN53 | 3 | indica | China |
| BN54 | 7 | japonica | China |
| BN55 | 3 | japonica | China |
| BN56 | 3 | indica | China |

| | | | |
|---|---|---|---|
| BN57 | 3 | intermediate type | China |
| BN58 | 3 | indica | China |
| BN59 | 3 | indica | China |
| BN60 | 1 | indica | China |
| BN61 | 3 | indica | China |
| BN62 | 1 | indica | China |
| BN63 | 5 | indica | China |
| BN64 | 3 | indica | China |
| BN65 | 3 | indica | China |
| BN66 | 3 | indica | China |
| BN67 | 3 | japonica | China |
| BN68 | 3 | japonica | China |
| BN69 | 3 | indica | India |
| BN70 | 3 | indica | China |
| BN71 | 3 | indica | China |
| BN72 | 5 | indica | China |
| BN73 | 3 | indica | China |
| BN74 | 5 | intermediate type | China |
| BN75 | 1 | japonica | China |
| BN76 | 1 | indica | China |
| BN77 | 5 | indica | China |
| BN78 | 5 | indica | China |
| BN79 | 5 | indica | China |
| BN80 | 5 | indica | China |
| BN81 | 3 | indica | China |
| BN82 | 3 | indica | China |
| BN83 | 5 | indica | China |
| BN84 | 5 | indica | China |
| BN85 | 7 | indica | China |
| BN86 | 5 | indica | China |
| BN87 | 1 | indica | China |
| BN88 | 9 | indica | China |
| BN89 | 9 | indica | China |
| BN90 | 7 | indica | China |
| BN91 | 7 | indica | China |
| BN92 | 5 | japonica | China |
| BN93 | 5 | indica | China |
| BN94 | 5 | japonica | China |
| BN95 | 3 | indica | Phillipines |
| BN96 | 3 | indica | China |
| BN97 | 7 | indica | China |
| BN98 | 7 | japonica | China |
| BN99 | 5 | indica | China |
| BN100 | 5 | indica | China |

| | | | |
|---|---|---|---|
| BN101 | 3 | indica | China |
| BN102 | 3 | japonica | China |
| BN103 | 7 | indica | China |
| BN104 | 7 | indica | China |
| BN105 | 5 | indica | China |
| BN106 | 1 | japonica | China |
| BN107 | 5 | indica | China |
| BN108 | 1 | japonica | China |
| BN109 | 3 | indica | China |
| BN110 | 5 | indica | China |
| BN111 | 3 | indica | China |
| BN112 | 5 | japonica | China |
| BN113 | 7 | japonica | China |
| BN114 | 3 | indica | China |
| BN115 | 1 | japonica | North Korea |
| BN116 | 1 | japonica | Japan |
| BN117 | 1 | japonica | Japan |
| BN118 | 7 | japonica | Russia |
| BN119 | 1 | japonica | Italy |
| BN120 | 7 | indica | Indonesia |
| BN121 | 3 | indica | Bangladesh |
| BN122 | 3 | japonica | France |
| BN123 | 5 | indica | Columbia |
| BN124 | 3 | japonica | Japan |
| BN125 | 5 | japonica | Japan |
| BN126 | 3 | indica | Vietnam |
| BN127 | 1 | indica | Laos |
| BN128 | 3 | indica | India |
| BN129 | 5 | intermediate type | Nigeria |
| BN130 | 7 | intermediate type | United States |
| BN131 | 1 | indica | Cuba |
| BN132 | 5 | indica | India |
| BN133 | 5 | japonica | China |
| BN134 | 5 | indica | China |
| BN135 | 3 | japonica | China |
| BN136 | 5 | indica | China |
| BN137 | 5 | indica | China |
| BN138 | 7 | japonica | China |
| BN139 | 5 | japonica | China |
| BN140 | 7 | indica | China |
| BN141 | 5 | indica | China |
| BN142 | 3 | indica | China |
| BN143 | 5 | indica | China |
| BN144 | 5 | indica | China |

| | | | |
|---|---|---|---|
| BN145 | 5 | indica | China |
| BN146 | 5 | indica | China |
| BN147 | 1 | indica | Phillipines |
| BN148 | 3 | indica | China |
| BN149 | 5 | japonica | China |
| BN150 | 5 | indica | China |
| BN151 | 5 | japonica | China |
| BN152 | 5 | indica | China |
| BN153 | 9 | japonica | China |
| BN154 | 9 | indica | China |
| BN155 | 7 | indica | China |
| BN156 | 5 | indica | China |
| BN157 | 5 | indica | China |
| BN158 | 5 | indica | China |
| BN159 | 7 | japonica | China |
| BN160 | 9 | indica | China |
| BN161 | 5 | indica | China |
| BN162 | 7 | japonica | China |
| BN163 | 5 | japonica | China |
| BN164 | 7 | indica | China |
| BN165 | 1 | indica | China |
| BN166 | 5 | indica | China |
| BN167 | 7 | indica | China |
| BN168 | 7 | indica | China |
| BN169 | 5 | japonica | United States |
| BN170 | 5 | indica | China |
| BN171 | 7 | indica | China |
| BN172 | 9 | indica | China |
| BN173 | 5 | indica | China |
| BN174 | 9 | indica | Thailand |
| BN175 | 7 | indica | China |
| BN176 | 7 | indica | China |
| BN177 | 5 | japonica | China |
| BN178 | 7 | indica | China |
| BN179 | 7 | indica | Phillipines |
| BN180 | 9 | intermediate type | China |
| BN181 | 3 | indica | China |
| BN182 | 3 | japonica | Japan |
| BN183 | 7 | indica | Phillipines |
| BN184 | 5 | indica | China |
| BN185 | 9 | japonica | China |
| BN186 | 1 | indica | Phillipines |
| BN187 | 7 | japonica | Japan |
| BN188 | 3 | japonica | China |

| | | | |
|---|---|---|---|
| BN189 | 5 | indica | Vietnam |
| BN190 | 1 | indica | China |
| BN191 | 3 | indica | China |

Num, number; Ori, origination

**Supplementary Table S2** | Salt tolerance evolution score (SES) difference of 191 accession population.

| Group | Num. | SD | Mean±SE | CV |
|---|---|---|---|---|
| all | 191 | 2.01 | 4.71±0.15 | 0.43 |
| indica | 129 | 1.98 | 4.74±0.17 | 0.42 |
| japonica | 57 | 2.07 | 4.54±0.27 | 0.46 |
| intermediate type | 5 | 2.28 | 5.80±1.02 | 0.39 |

SD, standard deviation; SE, standard error; CV, coefficient of variation

**Supplementary Table S3 |** Summary of candidate genes in all species GWAS.

| Num. | Gene ID | Gene Name | Description |
|---|---|---|---|
| 1 | LOC_Os01g62130 | | ZOS1-14 - C2H2 zinc finger protein, expressed |
| 2 | LOC_Os01g62190 | *ZFP179* | ZOS1-15 - C2H2 zinc finger protein, expressed |
| 3 | LOC_Os01g62200 | *OsDSR2* | domain of unknown function DUF966 domain containing protein, expressed |
| 4 | LOC_Os01g62410 | *OsMYB3R-2* | MYB family transcription factor putative, expressed |
| 5 | LOC_Os01g62430 | *OsERG1* | C2 domain containing protein putative, expressed |
| 6 | LOC_Os01g62460 | | ZOS1-16 - C2H2 zinc finger protein, expressed |
| 7 | LOC_Os01g62510 | | WRKY119, expressed |
| 8 | LOC_Os01g62514 | | WRKY56, expressed |
| 9 | LOC_Os03g12940 | | OsFBO13 - F-box and other domain containing protein, expressed |
| 10 | LOC_Os03g13310 | | MYB family transcription factor putative, expressed |
| 11 | LOC_Os03g13400 | *LPA1; OsIDD14* | ZOS3-06 - C2H2 zinc finger protein, expressed |
| 12 | LOC_Os03g13600 | | ZOS3-07 - C2H2 zinc finger protein, expressed |
| 13 | LOC_Os03g44740 | | cytochrome P450 putative, expressed |

| | | | |
|---|---|---|---|
| 14 | LOC_Os03g44800 | | DUF593 domain containing protein, expressed |
| 15 | LOC_Os03g44920 | | OsFBX98 - F-box domain containing protein, expressed |
| 16 | LOC_Os03g44950 | | LTPL91 - Protease inhibitor/seed storage/LTP family protein precursor, expressed |
| 17 | LOC_Os03g44980 | | OsFBX99 - F-box domain containing protein, expressed |
| 18 | LOC_Os03g45150 | | LTPL93 - Protease inhibitor/seed storage/LTP family protein precursor, expressed |
| 19 | LOC_Os04g51800 | | MYB protein putative, expressed |
| 20 | LOC_Os04g51820 | *OsHKT1; 1* | High-Affinity Potassium Transporter |
| 21 | LOC_Os04g51830 | *OsHKT1; 4* | OsHKT1;4 - Na+ transporter, expressed |
| 22 | LOC_Os04g52090 | *OsAP2-39* | AP2 domain containing protein, expressed |
| 23 | LOC_Os05g26940 | | CAMK_KIN1/SNF1/Nim1_like.23 - CAMK includes calcium/calmodulin depedent protein kinases, expressed |
| 24 | LOC_Os05g27550 | | OsFBX166 - F-box domain containing protein, expressed |
| 25 | LOC_Os06g08250 | | zinc finger family protein putative, expressed |
| 26 | LOC_Os06g08290 | *AID1* | MYB family transcription factor putative, expressed |
| 27 | LOC_Os06g08340 | *OsWR4; OsERF2* | AP2 domain containing protein, expressed |
| 28 | LOC_Os06g08570 | | PPR repeat domain containing protein putative, expressed |
| 29 | LOC_Os09g08920 | | cytochrome P450 putative, expressed |
| 30 | LOC_Os09g08990 | | cytochrome P450 putative, expressed |
| 31 | LOC_Os09g08620 | | OsFBX309 - F-box domain containing protein, expressed |
| 32 | LOC_Os12g41270 | | cysteine-rich receptor-like protein kinase 20 precursor putative, expressed |
| 33 | LOC_Os12g41300 | | OsFBX462 - F-box domain containing protein, expressed |
| 34 | LOC_Os12g41450 | | F-box domain containing protein, expressed |
| 35 | LOC_Os12g41490 | | cysteine-rich receptor-like protein kinase 8 precursor putative, expressed |
| 36 | LOC_Os12g41510 | | cysteine-rich receptor-like protein kinase 31 precursor putative, expressed |
| 37 | LOC_Os12g41520 | | cysteine-rich repeat secretory protein 26 precursor putative, expressed |
| 38 | LOC_Os12g41530 | | cysteine-rich receptor-like protein kinase 8 precursor putative, expressed |
| 39 | LOC_Os12g41620 | | WD domain G-beta repeat domain containing protein, expressed |
| 40 | LOC_Os12g41630 | | OsFBX463 - F-box domain containing protein, expressed |
| 41 | LOC_Os12g41680 | | No apical meristem protein putative, expressed |
| 42 | LOC_Os12g41700 | *OMTN3* | LSD1 zinc finger domain containing protein, expressed |

**Supplementary Table S4 |** Summary of candidate genes in indica species GWAS.

| Num. | Gene ID | Gene name | Description |
|---|---|---|---|
| 1 | LOC_Os01g38110 | | cytochrome P450 putative, expressed |
| 2 | LOC_Os01g40260 | *OsWRKY77* | WRKY77, expressed |

| | | | |
|---|---|---|---|
| 3 | LOC_Os01g40430 | *OsWRKY27* | WRKY27, expressed |
| 4 | LOC_Os01g40530 | | hsp20/alpha crystallin family protein, putative, expressed |
| 5 | LOC_Os01g40540 | | lectin receptor-type protein kinase putative, expressed |
| 6 | LOC_Os01g40550 | | hsp20/alpha crystallin family protein, putative, expressed |
| 7 | LOC_Os03g12940 | | OsFBO13 - F-box and other domain containing protein, expressed |
| 8 | LOC_Os03g13310 | | MYB family transcription factor putative, expressed |
| 9 | LOC_Os03g13400 | *LPA1; OsIDD14* | ZOS3-06 - C2H2 zinc finger protein, expressed |
| 10 | LOC_Os03g13600 | | ZOS3-07 - C2H2 zinc finger protein, expressed |
| 11 | LOC_Os03g27280 | *OsSAPK1* | CAMK_CAMK_like.19 - CAMK includes calcium/calmodulin depedent protein kinases, expressed |
| 12 | LOC_Os03g27460 | | heat shock protein DnaJ putative, expressed |
| 13 | LOC_Os04g51800 | | MYB protein putative, expressed |
| 14 | LOC_Os04g51820 | *OsHKT1; 1* | High-Affinity Potassium Transporter |
| 15 | LOC_Os04g52030 | | zinc finger domain LSD1 subclass family protein, expressed |
| 16 | LOC_Os04g52090 | *OsAP2-39* | AP2 domain containing protein, expressed |
| 17 | LOC_Os04g52120 | *OsHAK15* | potassium transporter putative, expressed |
| 18 | LOC_Os04g52290 | | PPR repeat domain containing protein putative, expressed |
| 19 | LOC_Os05g26940 | | CAMK_KIN1/SNF1/Nim1_like.23 - CAMK includes calcium/calmodulin depedent protein kinases, expressed |
| 20 | LOC_Os05g27550 | | OsFBX166 - F-box domain containing protein, expressed |
| 21 | LOC_Os06g08250 | | zinc finger family protein putative, expressed |
| 22 | LOC_Os06g08290 | *AID1* | MYB family transcription factor putative, expressed |
| 23 | LOC_Os06g08340 | *OsWR4; OsERF2* | AP2 domain containing protein, expressed |
| 24 | LOC_Os06g08570 | | PPR repeat domain containing protein putative, expressed |
| 25 | LOC_Os06g08360 | | ethylene-responsive element-binding protein putative, expressed |
| 26 | LOC_Os07g19210 | | cytochrome P450 putative, expressed |
| 27 | LOC_Os09g07780 | | pentatricopeptide repeat protein PPR986-12 putative, expressed |
| 28 | LOC_Os09g16510 | | WRKY74, expressed |
| 29 | LOC_Os09g16810 | | OsFBX317 - F-box domain containing protein, expressed |
| 30 | LOC_Os12g27790 | | OsFBX443 - F-box domain containing protein, expressed |
| 31 | LOC_Os12g27810 | | OsFBX444 - F-box domain containing protein, expressed |
| 32 | LOC_Os12g41160 | | IQ calmodulin-binding motif family protein putative, expressed |
| 33 | LOC_Os12g41270 | | cysteine-rich receptor-like protein kinase 20 precursor putative, expressed |
| 34 | LOC_Os12g41300 | | OsFBX462 - F-box domain containing protein, expressed |
| 35 | LOC_Os12g41450 | | F-box domain containing protein, expressed |
| 36 | LOC_Os12g41490 | | cysteine-rich receptor-like protein kinase 8 precursor putative, expressed |
| 37 | LOC_Os12g41510 | | cysteine-rich receptor-like protein kinase 31 precursor putative, expressed |
| 38 | LOC_Os12g41520 | | cysteine-rich repeat secretory protein 26 precursor putative, expressed |
| 39 | LOC_Os12g41530 | | cysteine-rich receptor-like protein kinase 8 precursor putative, expressed |

| | | | |
|---|---|---|---|
| 40 | LOC_Os12g41620 | | WD domain G-beta repeat domain containing protein, expressed |
| 41 | LOC_Os12g41630 | | OsFBX463 - F-box domain containing protein, expressed |
| 42 | LOC_Os12g41680 | *OMTN3* | No apical meristem protein putative, expressed |

**Supplementary Table S5 |** Summary of candidate genes in japonica species GWAS.

| Num. | Gene ID | Gene name | Description |
|---|---|---|---|
| 1 | LOC_Os01g09640 | | MYB transcription factor putative, expressed |
| 2 | LOC_Os01g09790 | | IQ calmodulin-binding motif domain containing protein, expressed |
| 3 | LOC_Os01g09850 | *OsIDD2* | ZOS1-04 - C2H2 zinc finger protein, expressed |
| 4 | LOC_Os01g10040 | *D2; CYP90D2* | cytochrome P450 putative, expressed |
| 5 | LOC_Os01g21940 | | WD domain G-beta repeat domain containing protein, expressed |
| 6 | LOC_Os01g36600 | | PPR repeat domain containing protein putative, expressed |
| 7 | LOC_Os01g36940 | | OsFBX13 - F-box domain containing protein, expressed |
| 8 | LOC_Os01g40430 | *OsWRKY27* | WRKY27, expressed |
| 9 | LOC_Os01g48130 | | no apical meristem protein putative, expressed |
| 10 | LOC_Os01g48250 | | OsFBDUF5 - F-box and DUF domain containing protein, expressed |
| 11 | LOC_Os01g48370 | | OsFBT1 - F-box and tubby domain containing protein, expressed |
| 12 | LOC_Os01g48380 | *ALS3* | PPR repeat domain containing protein putative, expressed |
| 13 | LOC_Os01g48446 | | no apical meristem protein putative, expressed |
| 14 | LOC_Os01g51690 | *OsWRKY26* | WRKY26, expressed |
| 15 | LOC_Os01g51840 | | IQ calmodulin-binding motif family protein putative, expressed |
| 16 | LOC_Os01g52640 | | suppressor of phythchrome A putative, expressed |
| 17 | LOC_Os01g52790 | | cytochrome P450 72A1 putative, expressed |
| 18 | LOC_Os01g52800 | | cytochrome P450 72A1 putative, expressed |
| 19 | LOC_Os01g52970 | | OsFBX22 - F-box domain containing protein, expressed |
| 20 | LOC_Os01g52980 | | OsFBX23 - F-box domain containing protein, expressed |
| 21 | LOC_Os01g53040 | *OsWRKY14* | WRKY14, expressed |
| 22 | LOC_Os01g54380 | | PPR repeat domain containing protein putative, expressed |
| 23 | LOC_Os01g61080 | *OsWRKY24* | WRKY24, expressed |
| 24 | LOC_Os01g61590 | | CAMK_CAMK_like.1 - CAMK includes calcium/calmodulin depedent protein kinases, expressed |
| 25 | LOC_Os01g65370 | | MYB family transcription factor putative, expressed |
| 26 | LOC_Os01g67970 | *JMJ705* | ZOS1-20 - C2H2 zinc finger protein, expressed |
| 27 | LOC_Os02g08140 | | CAMK_KIN1/SNF1/Nim1_like.14 - CAMK includes calcium/calmodulin depedent protein kinases, expressed |
| 28 | LOC_Os02g14490 | | MYB family transcription factor putative, expressed |
| 29 | LOC_Os02g14790 | | WD domain G-beta repeat domain containing protein, expressed |
| 30 | LOC_Os02g18820 | | WD domain G-beta repeat domain containing protein, expressed |
| 31 | LOC_Os02g19180 | | ZOS2-06 - C2H2 zinc finger protein, expressed |
| 32 | LOC_Os02g19200 | | OsFBX46 - F-box domain containing protein, expressed |

| | | | |
|---|---|---|---|
| 33 | LOC_Os02g21230 | | F-box domain containing protein, expressed |
| 34 | LOC_Os02g21490 | | WD domain G-beta repeat domain containing protein, expressed |
| 35 | LOC_Os02g21580 | | PPR repeat containing protein, expressed |
| 36 | LOC_Os02g21810 | | cytochrome P450 51 putative, expressed |
| 37 | LOC_Os02g22020 | *NIGT1* | MYB family transcription factor putative, expressed |
| 38 | LOC_Os02g36030 | | cytochrome P450 putative, expressed |
| 39 | LOC_Os02g36070 | | cytochrome P450 putative, expressed |
| 40 | LOC_Os02g36110 | *CYP76M7* | cytochrome P450 putative, expressed |
| 41 | LOC_Os02g36150 | | cytochrome P450 putative, expressed |
| 42 | LOC_Os02g36190 | | cytochrome P450 putative, expressed |
| 43 | LOC_Os02g36280 | | cytochrome P450 putative, expressed |
| 44 | LOC_Os02g36360 | | ZOS2-11 - C2H2 zinc finger protein, expressed |
| 45 | LOC_Os02g36880 | | No apical meristem protein putative, expressed |
| 46 | LOC_Os02g42850 | | MYB family transcription factor putative, expressed |
| 47 | LOC_Os02g42870 | | MYB family transcription factor putative, expressed |
| 48 | LOC_Os02g42970 | | NAC domain containing protein putative, expressed |
| 49 | LOC_Os02g43080 | | PPR repeat domain containing protein putative, expressed |
| 50 | LOC_Os02g49040 | | CAMK_CAMK_like_unique.1 - CAMK includes calcium/calmodulin depedent protein kinases, expressed |
| 51 | LOC_Os02g49090 | | WD domain G-beta repeat domain containing protein, expressed |
| 52 | LOC_Os02g49250 | | myb-like DNA-binding domain containing protein, expressed |
| 53 | LOC_Os02g55050 | | OsFBX64 - F-box domain containing protein, expressed |
| 54 | LOC_Os02g55380 | *OsWR3* | AP2 domain containing protein, expressed |
| 55 | LOC_Os03g04530 | | cytochrome P450 putative, expressed |
| 56 | LOC_Os03g04630 | | cytochrome P450 protein putative, expressed |
| 57 | LOC_Os03g04640 | | cytochrome P450 protein putative, expressed |
| 58 | LOC_Os03g04650 | | cytochrome P450 protein putative, expressed |
| 59 | LOC_Os03g04660 | *WSL5/CYP96B5* | cytochrome P450 86A1 putative, expressed |
| 60 | LOC_Os03g04680 | *OsCYP96B4; OsDSS1* | cytochrome P450 putative, expressed |
| 61 | LOC_Os03g04900 | | MYB family transcription factor putative, expressed |
| 62 | LOC_Os03g06370 | *OsNBL3* | PPR repeat domain containing protein putative, expressed |
| 63 | LOC_Os03g10040 | | OsFBX77 - F-box domain containing protein, expressed |
| 64 | LOC_Os03g10140 | | ZOS3-04 - C2H2 zinc finger protein, expressed |
| 65 | LOC_Os03g10420 | | PPR repeat containing protein, expressed |
| 66 | LOC_Os03g18480 | *OsTDF1* | MYB family transcription factor putative, expressed |
| 67 | LOC_Os03g18620 | | PPR repeat domain containing protein putative, expressed |
| 68 | LOC_Os03g20500 | | OsFBX82 - F-box domain containing protein, expressed |
| 69 | LOC_Os03g20550 | *OsWRKY55; OsWRKY31* | WRKY55, expressed |
| 70 | LOC_Os03g20900 | | Myb transcription factor putative, expressed |
| 71 | LOC_Os03g21030 | | no apical meristem protein putative, expressed |
| 72 | LOC_Os03g42630 | | No apical meristem protein putative, expressed |
| 73 | LOC_Os03g43060 | | OsFBX96 - F-box domain containing protein, expressed |

| | | | |
|---|---|---|---|
| 74 | LOC_Os03g49990 | *SLR1; OsGAI* | GRAS family transcription factor domain containing protein, expressed |
| 75 | LOC_Os03g53050 | *OsWRKY87* | WRKY121, expressed |
| 76 | LOC_Os03g53060 | | cytochrome P450 putative, expressed |
| 77 | LOC_Os03g56580 | | no apical meristem protein putative, expressed |
| 78 | LOC_Os03g56960 | | PPR repeat domain containing protein putative, expressed |
| 79 | LOC_Os03g59730 | | No apical meristem protein putative, expressed |
| 80 | LOC_Os03g60080 | *SNAC1; OsNAC9* | NAC domain-containing protein 67 putative, expressed |
| 81 | LOC_Os03g60120 | | AP2 domain containing protein, expressed |
| 82 | LOC_Os03g60430 | *OsIDS1* | AP2 domain containing protein, expressed |
| 83 | LOC_Os03g60540 | | ZOS3-20 - C2H2 zinc finger protein, expressed |
| 84 | LOC_Os03g60560 | *ZFP182; ZOS3-21* | ZOS3-21 - C2H2 zinc finger protein, expressed |
| 85 | LOC_Os03g60570 | *ZFP15* | ZOS3-22 - C2H2 zinc finger protein, expressed |
| 86 | LOC_Os03g60910 | | PPR repeat domain containing protein putative, expressed |
| 87 | LOC_Os04g33370 | | cytochrome P450 putative, expressed |
| 88 | LOC_Os04g33820 | | OsFBX132 - F-box domain containing protein, expressed |
| 89 | LOC_Os05g04210 | | MYB family transcription factor putative, expressed |
| 90 | LOC_Os05g04340 | | CGMC_GSK.6 - CGMC includes CDA MAPK GSK3 and CLKC kinases, expressed |
| 91 | LOC_Os05g04550 | | CAMK_KIN1/SNF1/Nim1_like.3 - CAMK includes calcium/calmodulin depedent protein kinases, expressed |
| 92 | LOC_Os05g04640 | *OsWRKY5* | WRKY5, expressed |
| 93 | LOC_Os05g14130 | | ZOS5-05 - C2H2 zinc finger protein, expressed |
| 94 | LOC_Os05g34310 | *OsNAC024* | no apical meristem protein putative, expressed |
| 95 | LOC_Os05g35010 | | cytochrome P450 putative, expressed |
| 96 | LOC_Os05g35170 | *IDEF2* | no apical meristem protein putative, expressed |
| 97 | LOC_Os05g35410 | *OsAKT2* | potassium channel AKT2/3, putative, expressed |
| 98 | LOC_Os05g35500 | *Osmyb1* | MYB family transcription factor putative, expressed |
| 99 | LOC_Os05g39090 | | CAMK_CAMK_like.29 - CAMK includes calcium/calmodulin depedent protein kinases, expressed |
| 100 | LOC_Os05g39590 | | AP2 domain containing protein, expressed |
| 101 | LOC_Os05g41760 | *MFS1* | AP2 domain containing protein, expressed |
| 102 | LOC_Os05g50560 | *OsMPK9* | CGMC_MAPKCMGC_2.9 - CGMC includes CDA MAPK GSK3 and CLKC kinases, expressed |
| 103 | LOC_Os05g50610 | *OsWRKY8* | WRKY8, expressed |
| 104 | LOC_Os05g50680 | | WRKY83, expressed |
| 105 | LOC_Os05g50700 | | WRKY111, expressed |
| 106 | LOC_Os05g50710 | *OsLEA5* | late embryogenesis abundant protein putative, expressed |
| 107 | LOC_Os05g50810 | | CAMK_CAMK_like.5 - CAMK includes calcium/calmodulin depedent protein kinases, expressed |
| 108 | LOC_Os05g50950 | | pentatricopeptide repeat protein PPR986-12 putative, expressed |
| 109 | LOC_Os05g51160 | | Myb transcription factor putative, expressed |
| 110 | LOC_Os06g19660 | | WD domain G-beta repeat domain containing protein, expressed |
| 111 | LOC_Os06g19670 | | OsFBX196 - F-box domain containing protein, expressed |

| | | | |
|---|---|---|---|
| 112 | LOC_Os06g19980 | | MYB family transcription factor putative, expressed |
| 113 | LOC_Os06g20020 | | ZOS6-03 - C2H2 zinc finger protein, expressed |
| 114 | LOC_Os06g47400 | | OsFBX204 - F-box domain containing protein, expressed |
| 115 | LOC_Os06g47570 | | PPR repeat containing protein, expressed |
| 116 | LOC_Os06g47590 | | AP2 domain containing protein, expressed |
| 117 | LOC_Os06g47840 | | ZOS6-08 - C2H2 zinc finger protein, expressed |
| 118 | LOC_Os06g47860 | *OsSIDP366* | expressed protein |
| 119 | LOC_Os07g08180 | | PPR repeat containing protein, expressed |
| 120 | LOC_Os07g08570 | | OsFBX218 - F-box domain containing protein, expressed |
| 121 | LOC_Os07g08860 | *OsSIK2* | S-domain receptor-like protein kinase putative, expressed |
| 122 | LOC_Os07g09000 | *OsPHF1* | WD domain G-beta repeat domain containing protein, expressed |
| 123 | LOC_Os07g09110 | | OsFBX219 - F-box domain containing protein, expressed |
| 124 | LOC_Os07g25370 | | myb-like DNA-binding domain containing protein, expressed |
| 125 | LOC_Os07g25440 | | WD domain G-beta repeat domain containing protein, expressed |
| 126 | LOC_Os07g25710 | *OsPHR2* | myb-like DNA-binding domain containing protein, expressed |
| 127 | LOC_Os07g37400 | *OsMsr9* | OsFBX257 - F-box domain containing protein, expressed |
| 128 | LOC_Os07g37920 | | no apical meristem protein putative, expressed |
| 129 | LOC_Os07g37970 | | cytochrome P450 51 putative, expressed |
| 130 | LOC_Os07g37980 | | cytochrome P450 51 putative, expressed |
| 131 | LOC_Os07g38030 | | GRAS family transcription factor domain containing protein, expressed |
| 132 | LOC_Os07g38120 | *OsCPK20* | CAMK_CAMK_like.34 - CAMK includes calcium/calmodulin depedent protein kinases, expressed |
| 133 | LOC_Os07g42510 | | AP2 domain containing protein, expressed |
| 134 | LOC_Os07g42770 | | CAMK_CAMK_like.35 - CAMK includes calcium/calmodulin depedent protein kinases, expressed |
| 135 | LOC_Os07g42880 | | PPR repeat containing protein, expressed |
| 136 | LOC_Os07g42940 | *OsSAPK2* | CAMK_CAMK_like.7 - CAMK includes calcium/calmodulin depedent protein kinases, expressed |
| 137 | LOC_Os08g04270 | *OsFIE2* | WD domain G-beta repeat domain containing protein, expressed |
| 138 | LOC_Os08g04290 | *OsFIE1; Epi-df* | WD domain G-beta repeat domain containing protein, expressed |
| 139 | LOC_Os08g04840 | | MYB family transcription factor putative, expressed |
| 140 | LOC_Os08g18150 | | WD domain G-beta repeat domain containing protein, expressed |
| 141 | LOC_Os08g18880 | | WD domain G-beta repeat domain containing protein, expressed |
| 142 | LOC_Os08g36790 | *TRAB1; OsbZIP66* | bZIP transcription factor putative, expressed |
| 143 | LOC_Os08g36860 | *OsABA8ox2* | cytochrome P450 putative, expressed |
| 144 | LOC_Os08g36920 | | AP2 domain containing protein, expressed |
| 145 | LOC_Os09g03500 | | ZOS9-01 - C2H2 zinc finger protein, expressed |
| 146 | LOC_Os09g10980 | | ZOS9-02 - C2H2 zinc finger protein, expressed |
| 147 | LOC_Os09g21260 | | cytochrome P450 domain containing protein, expressed |
| 148 | LOC_Os09g24560 | | No apical meristem protein putative, expressed |
| 149 | LOC_Os09g24800 | | MYB family transcription factor putative, expressed |
| 150 | LOC_Os09g24840 | | GASR10 - Gibberellin-regulated GASA/GAST/Snakin family protein precursor, expressed |

| | | | |
|---|---|---|---|
| 151 | LOC_Os09g26050 | | ZOS9-09 - C2H2 zinc finger protein, expressed |
| 152 | LOC_Os09g26100 | | ZOS9-10 - C2H2 zinc finger protein, expressed |
| 153 | LOC_Os09g26170 | | MYB family transcription factor putative, expressed |
| 154 | LOC_Os09g26200 | | ZOS9-11 - C2H2 zinc finger protein, expressed |
| 155 | LOC_Os09g26210 | | ZOS9-12 - C2H2 zinc finger protein, expressed |
| 156 | LOC_Os09g26400 | *OsDSG1* | zinc finger C3HC4 type domain containing protein, expressed |
| 157 | LOC_Os09g26420 | *OsBIERF1* | AP2 domain containing protein, expressed |
| 158 | LOC_Os09g26780 | *OsJAZ8* | zinc-finger protein putative, expressed |
| 159 | LOC_Os09g29170 | | CAMK_CAMK_like.38 - CAMK includes calcium/calmodulin depedent protein kinases, expressed |
| 160 | LOC_Os09g32910 | | OsFBX337 - F-box domain containing protein, expressed |
| 161 | LOC_Os09g32972 | | MYB protein putative, expressed |
| 162 | LOC_Os09g33490 | | no apical meristem protein putative, expressed |
| 163 | LOC_Os09g33910 | | CAMK_CAMK_like.39 - CAMK includes calcium/calmodulin depedent protein kinases, expressed |
| 164 | LOC_Os09g37949 | *OsRPK1* | serine/threonine-protein kinase SRPK1, putative, expressed |
| 165 | LOC_Os09g38000 | *YL3; OsNAC109* | no apical meristem protein putative, expressed |
| 166 | LOC_Os09g38010 | | no apical meristem protein putative, expressed |
| 167 | LOC_Os09g38340 | | ZOS9-17 - C2H2 zinc finger protein, expressed |
| 168 | LOC_Os10g38834 | *OsSWN4* | no apical meristem protein putative, expressed |
| 169 | LOC_Os10g38950 | *OsMPK6; OsMPK4* | CGMC_MAPKCMGC_2_ERK.14 - CGMC includes CDA MAPK GSK3 and CLKC kinases, expressed |
| 170 | LOC_Os10g41130 | | AP2 domain containing protein, expressed |
| 171 | LOC_Os10g41200 | *MYBS3* | MYB family transcription factor putative, expressed |
| 172 | LOC_Os10g41260 | | MYB family transcription factor putative, expressed |
| 173 | LOC_Os10g41330 | | AP2 domain containing protein, expressed |
| 174 | LOC_Os10g41400 | *OsMSRA4.1* | peptide methionine sulfoxide reductase putative, expressed |
| 175 | LOC_Os10g41490 | *OsSAPK3; REK* | CAMK_CAMK_like.41 - CAMK includes calcium/calmodulin depedent protein kinases, expressed |
| 176 | LOC_Os10g42760 | | PPR repeat domain containing protein putative, expressed |
| 177 | LOC_Os10g42850 | *OsWRKY2* | WRKY2, expressed |
| 178 | LOC_Os11g03110 | | GRAS family transcription factor domain containing protein, expressed |
| 179 | LOC_Os11g03300 | *OsNAC10; ONAC122* | NAC domain transcription factor putative, expressed |
| 180 | LOC_Os11g03310 | | no apical meristem protein putative, expressed |
| 181 | LOC_Os11g03370 | | no apical meristem protein putative, expressed |
| 182 | LOC_Os11g03440 | | myb-like DNA-binding domain containing protein putative, expressed |
| 183 | LOC_Os11g03540 | | AP2 domain containing protein, expressed |
| 184 | LOC_Os12g04480 | | cytochrome P450 putative, expressed |
| 185 | LOC_Os12g05440 | *CYP94C2b* | cytochrome P450 putative, expressed |
| 186 | LOC_Os12g12860 | *OSIPK* | CAMK_CAMK_like.46 - CAMK includes calcium/calmodulin depedent protein kinases, expressed |
| 187 | LOC_Os12g13130 | | ZOS12-03 - C2H2 zinc finger protein, expressed |

| | | | |
|---|---|---|---|
| 188 | LOC_Os12g21930 | | PPR repeat containing protein, expressed |
| 189 | LOC_Os12g38180 | *OsHSP23.7* | heat shock cognate 70 kDa protein 2 putative, expressed |
| 190 | LOC_Os12g38400 | *OsMYB91* | MYB family transcription factor putative, expressed |
| 191 | LOC_Os12g41630 | | OsFBX463 - F-box domain containing protein, expressed |

**Supplementary Table S6|** Summary of candidate genes observed in domestication region in Asian and African rice.

| Num. | Gene ID | Gene Name | Or_ind | Or_jap | Ob_Og |
|---|---|---|---|---|---|
| 1 | LOC_Os02g21810 | - | 1 | 1 | 1 |
| 2 | LOC_Os05g35170 | *IDEF2* | 1 | - | 1 |
| 3 | LOC_Os10g41130 | - | - | 1 | 1 |
| 4 | LOC_Os10g41200 | *MYBS3* | - | 1 | 1 |
| 5 | LOC_Os10g41260 | - | - | 1 | 1 |
| 6 | LOC_Os10g41330 | - | - | 1 | 1 |
| 7 | LOC_Os12g41680 | *OMTN3* | - | 1 | 1 |

1 represents the gene was observed in domestication region in Asian or African rice. Or, *O. rufipogon*; ind, *indica*; jap, *japonica*; Ob, *O. barthii*; Og, *O. glaberrima*.

**Supplementary Table S7|** Summary of the transcriptome data of GO classification at ST1 (93-11$_{ST0}$ vs. 93-11$_{ST100}$).

| GO Name | GO ID | P value | Differential genes | ORs value |
|---|---|---|---|---|
| extracellular region | GO:0005576 | 3.54 | 27 | 0.073 |
| cell wall | GO:0005618 | 2.29 | 31 | 0.058 |
| external encapsulating structure | GO:0030312 | 2.22 | 31 | 0.057 |
| thylakoid | GO:0009579 | 1.96 | 24 | 0.058 |
| membrane | GO:0016020 | 1.72 | 153 | 0.041 |
| response to abiotic stimulus | GO:0009628 | 11.65 | 124 | 0.071 |
| response to stress | GO:0006950 | 11.17 | 158 | 0.063 |
| response to chemical | GO:0042221 | 10.97 | 170 | 0.061 |
| response to endogenous stimulus | GO:0009719 | 7.82 | 100 | 0.067 |
| response to biotic stimulus | GO:0009607 | 4.29 | 63 | 0.064 |
| carbohydrate metabolic process | GO:0005975 | 3.81 | 34 | 0.076 |
| response to external stimulus | GO:0009605 | 3.57 | 77 | 0.057 |
| response to light stimulus | GO:0009416 | 3.17 | 41 | 0.066 |
| secondary metabolic process | GO:0019748 | 2.14 | 21 | 0.070 |

**Supplementary Table S8|** Summary of the transcriptome data of GO classification at ST2 (PA64$_{SST0}$ vs. PA64$_{SST100}$).

| GO Name | GO ID | P value | Differential gene | ORs value |
|---|---|---|---|---|
| transporter activity | GO:0005215 | 2.80 | 94 | 0.112 |
| carbohydrate binding | GO:0030246 | 1.37 | 11 | 0.149 |
| membrane | GO:0016020 | 7.38 | 370 | 0.098 |
| cell wall | GO:0005618 | 5.60 | 73 | 0.135 |
| external encapsulating structure | GO:0030312 | 5.43 | 73 | 0.134 |
| plasma membrane | GO:0005886 | 4.16 | 212 | 0.099 |
| extracellular region | GO:0005576 | 3.16 | 47 | 0.127 |
| thylakoid | GO:0009579 | 2.91 | 50 | 0.122 |
| vacuole | GO:0005773 | 1.56 | 76 | 0.098 |
| response to chemical | GO:0042221 | 14.29 | 334 | 0.120 |
| response to abiotic stimulus | GO:0009628 | 9.28 | 215 | 0.123 |
| response to endogenous stimulus | GO:0009719 | 7.02 | 180 | 0.120 |
| response to stress | GO:0006950 | 6.52 | 272 | 0.109 |
| response to light stimulus | GO:0009416 | 5.92 | 87 | 0.139 |
| response to external stimulus | GO:0009605 | 5.67 | 159 | 0.118 |
| secondary metabolic process | GO:0019748 | 4.64 | 47 | 0.156 |
| response to biotic stimulus | GO:0009607 | 4.50 | 118 | 0.120 |
| lipid metabolic process | GO:0006629 | 3.84 | 83 | 0.124 |
| post-embryonic development | GO:0009791 | 3.74 | 149 | 0.110 |
| multicellular organism development | GO:0007275 | 2.48 | 224 | 0.098 |
| anatomical structure development | GO:0048856 | 1.99 | 252 | 0.095 |
| cellular homeostasis | GO:0019725 | 1.85 | 25 | 0.133 |
| generation of precursor metabolites and energy | GO:0006091 | 1.54 | 31 | 0.119 |
| reproduction | GO:0000003 | 1.47 | 137 | 0.097 |
